# Measurements of Aerodynamic Interference of a Hybrid Aircraft with Multirotor Propulsion †

**DOI:** 10.3390/s20123360

**Published:** 2020-06-13

**Authors:** Zbigniew Czyż, Mirosław Wendeker

**Affiliations:** 1 Aeronautics Faculty, Military University of Aviation, 35 Dywizjonu 303 St., 08-521 Dęblin, Poland; 2 Department of Thermodynamics, Fluid Mechanics and Aviation Propulsion Systems, Faculty of Mechanical Engineering, Lublin University of Technology, 36 Nadbystrzycka St., 20-618 Lublin, Poland; m.wendeker@pollub.pl

**Keywords:** measurements of aerodynamic forces, wind tunnel research, aerodynamic interference, autogyros, multirotors

## Abstract

This article deals with the phenomenon of aerodynamic interference occurring in the innovative hybrid system of multirotor aircraft propulsion. The approach to aerodynamics requires a determination of the impact of active sources of lift and thrust upon the aircraft aerodynamic characteristics. The hybrid propulsion unit, composed of a conventional multirotor source of thrust as well as lift in the form of the main rotor and a pusher, was equipped with an additional propeller drive unit. The tests were conducted in a continuous-flow low speed wind tunnel with an open measuring space, 1.5 m in diameter and 2.0 m long. Force testing made it possible to develop aerodynamic characteristics as well as defining aerodynamic characteristics and defining the field of speed for the considered design configurations. Our investigations enabled us to analyze the results in terms of a mutual impact of particular components of the research object and the area of impact of active elements present in a common flow.

## 1. Introduction

A gyroplane is a type of a rotorcraft that was originally developed in Spain in 1920. The first successful flight of the Cierva C-4 rotorcraft took place on 9 January 1923 [1,2,3,4]. A demonstration of a successful flight proved that it can be a useful and practical machine [4,5]. After a short time, however, the research was moved to Great Britain and the British military forces became interested in this type of aircraft. Soon also other countries gained the right to use patents to manufacture their own gyroplanes in their own gyroplane companies. During this time, the research was conducted in Great Britain by Cierva Autogiro Company (Juan de la Cierva and his team) as well as in the United States by the American Autogiro Company (Harold F. Pitcairn and his team). By 1937, both teams became the leading aircraft manufacturers. According to the authors of work [5], the emergence of gyroplanes and the conducted investigations in the 1920s and 1930s paved the way for the development of helicopters in the 1940s. A number of technical in-flight problems related to rotary wings (rotor blades), especially in relation to the solutions proposed by Juan de la Cierva, were noticed at that time, e.g., gimbal mount of the main rotor blades. As helicopters became more popular than gyroplanes in the years 1940–1980, the latter were affected by the so-called “dead period”, mainly because of the war, politics and history, but not due to technical issues of the machines [3,6,7]. In recent years, however, there has been a revival of interest in this type of flying machine, both as an entertainment aircraft as well as a cheaper alternative to helicopters. Autogyros manufacturers sell their machines not only to commercial and military operators but primarily to individuals who use these machines for hobby flying.

The work [8] presents the results of comprehensive research into gyroplane flight dynamics. The authors developed and approved modern flight simulation of this type of aircraft in relation to the assumed parameters, and their model was validated. After conducting simulation tests, it was found that the overall characteristics of gyroplane stability are a combination of flight models for a helicopter and an airplane. The results presented in work [6] are one of the most significant achievements in the field of aerodynamics of gyroplanes. The description of these results has also been presented in [8]. The characteristics of flight dynamics of gyroplanes were discussed in works [7,9,10,11]. The work [12] is a numerical analysis in order to investigate a new concept of a gyroplane in a progressive flight. The aircraft was a combination of an airframe and a gyroplane. The TSM method (Transient Simulation Method) was used for an analysis of performance of the autorotating rotor. In the initial phase, the calculations of the fuselage were made in order to identify the variability of aerodynamic properties depending on the flight speed. Under certain conditions (speed, pitch angle of the rotor and blades), it was possible to determine quasi-static states of autorotation, as well as observing their impact on the operation of the rotor. The results of the analysis showed a significant effect of the fuselage on the aerodynamic properties of an aircraft. It turned out that the unfavorable limitation of progressive speed largely depends on the autorotation performance of a rotor.

The paper [13] focuses on the selection and optimization of key parameters of the aircraft fleet used in Small Air Transport. An important feature of the Small Air Transport system is personalization, which is the flexible adaptation of the transport process to the customer’s requirements and capabilities. One of the desirable features of the gyroplane is to perform a vertical take-off and even a hover. Another important criterion is achieving high cruising speeds which do not interfere with the limitations of noise emissions from the main rotor. An implementation of the above assumptions makes it possible to build a rotorcraft whose capabilities will exceed other helicopters as well as being competitive to aircraft. A logical and practical solution seems to combine the advantages of gyroplanes and increasingly popular multicopters and also to design a quiet, fast VTOL (Vertical Take Off and Landing) flying machine. Presumably it will prove economical to use, safe and able to perform tasks previously assigned to conventional aircraft. Multicopter engines, during a transition from a hover to a horizontal flight with increasing speed, increase power demand. There is a possibility to reduce energy consumption by offloading the propulsion unit during a flight by a gyroplane rotor, operating in autorotation.

For economic reasons, multirotor propulsion units are not beneficial since during a progressive motion, their demand for power grows. The aerodynamic phenomena occurring during a flight of these aircrafts have been described in detail in works [14,15,16,17]. In order to explain a relationship between the thrust, angle of attack and airspeeds, for constant power it is necessary to use the relationships cited in the above-mentioned works. The rotor generates thrust by accelerating the air that flows through it. On the basis of an analysis of momentum as a result of generating the thrust force *T_h_*, induced speed *v_h_* is generated, as described by a relationship (1):

(1) $$v_{h}=\sqrt{\frac{T_{h}}{2\rho A}}$$

where:

*A*—rotor disc area [m^2^],

*ρ*—air density [kg/m^3^].

For an ideal aircraft, the speed υ_h_ may be dependent on the flight speed *v_i_* in accordance with Formula (2):

(2) $$v_{i}=\sqrt{\frac{v_{h}^{2}}{{\left(v_{\infty }cos\alpha \right)}^{2}+{\left(v_{i}-v_{\infty }sin\alpha \right)}^{2}}}$$

where:

*α*—angle of attack between the disc rotor plane and the vector of speed of an undisturbed stream of flow on the assumption that positive values are achieved along with an increased pitch angle of an aircraft.

On the basis of a relationship describing *v_i_* it is possible to calculate ideal thrust *T*, for input power *P*, as (3):

(3) $$T=\frac{P}{v_{i}-v_{\infty }sin\alpha }$$

In dependence (3), the denominator is the speed of flow through the rotor. The input power required to achieve a nominal thrust *T_h_* can be calculated as (4):

(4) $$P=\frac{T_{h}^{3/2}}{\sqrt{2\rho A}}$$

A combination of these equations allows for determining the ratio of thrust occurring during a progressive flight to the thrust force during a hover.

The aircraft concept using rotor autorotation at high speeds is difficult to develop in an analytical way, even though it was proposed a long time ago [18,19]. The situation looks much better in relation to helicopters [20]. The works [3,20] show the basics of the fundamentals of autorotation with regard to helicopters. In [3], the lift force of the rotor in autorotation is described by a dependence (5). For a given overload during a flight equal to 1 G, thrust must be equal to the mass of the gyroplane [21].

(5) $$T=\frac{1}{2}\rho aNc\Omega^{2}R^{3}\left(\frac{\theta }{3}+\frac{\lambda }{2}\right)$$

where:

*ρ*—air density [kg/m^3^],

*a*—two-dimensional lift slope factor [–],

*N*—number of blades [–],

*c*—blade chord [m];

*R*—rotor radius [m],

*θ*—blade pitch angle (the constant value is assumed on the rotor radius) [°],

*λ*—inflow factor [–].

In order to determine the angular velocity of rotor Ω and the factor *λ*, it is necessary to take into consideration the torque of rotor *M_T_* (*C_D_*_0_ is a drag force coefficient of an airfoil):

(6) $$M_{T}=\frac{1}{2}\rho aNc\Omega^{2}R^{4}\left[\frac{C_{DO}}{4}-a\lambda \left(\frac{\theta }{3}+\frac{\lambda }{2}\right)\right]$$

Due to the fact that the gyroplane rotor is not propelled, the torque must be equal to 0:

(7) $$M_{T}=0$$

In this case, the inflow factor may be solved from equation:

(8) $$\lambda =-\frac{\theta }{3}+\frac{1}{a}\sqrt{{\left(\frac{a\theta }{3}\right)}^{2}+a\frac{C_{DO}}{2}}$$

An introduction of *λ* into (5) along with the rotor solidity *σ* (*c* is a blade chord at 0.7 *R*):

(9) $$\sigma_{0.7}=\frac{cb}{\pi R}$$

The angular velocity Ω of the gyroplane rotor generates a lifting force equal to the weight of an aircraft, resulting from:

(10) $$\Omega =\frac{1}{R}\sqrt{\frac{W}{\frac{\sigma_{0.7}a}{2}}\left(\frac{\theta }{3}+\frac{\lambda }{2}\right)\pi R^{2}\rho }$$

Vertical descent speed of the gyroplane and the coefficient of thrust can be described by the following equations:

(11) $$v_{ver}=\sqrt{\frac{W}{2\pi R^{2}\rho \frac{C_{T}}{4\lambda^{2}\left(\frac{C_{T}}{2\lambda^{2}}+1\right)}}}$$

(12) $$C_{T}=\frac{\sigma_{0.7}a}{2}\left(\frac{\theta }{3}+\frac{\lambda }{2}\right)$$

In helicopters, the autorotation maneuver is used to land in the event of a loss of engine power. During the maneuver, the collected input data are used to control the airspeed while reducing the altitude and exploiting the energy stored in the main rotor. Apart from the strategy of autorotation landing directly piloted, several automatic strategies have also been developed. The majority of them contain a simple system of flight control using the concepts of simplified aircraft dynamics so as to land in a pre-defined area. It is possible to access a range of investigations with regard to optimal autorotation [22]. The most recent works also suggest the results of route planning [23,24] taking into account terrain layout [25], ground obstacles [26,27] and the so-called complex detection [28]. These findings have been used by pilots to assist their decision or to execute a fully autonomous flight. In the latter case, an autonomous landing strategy during a real flight has never been shown for a manned helicopter landing.

A general theory about the aerodynamics of an autorotation rotor was based on the theory of blade elements (BET) by Glauert and Stepniewski [29,30,31], who presented the classic form of induced speed, and by NASA and John B. Wheatley [32,33,34,35,36,37], who carried out a complete experiment on the gyroplane PCA–2 in a real scale, reaching the lift and thrust coefficients. In the theory of the blade element, the ratio of momentum balance has been used to calculate thrust in the rotor plane, providing general characteristics of the rotor flow [38].

The phenomenon of aerodynamic interference was defined in work [33] as a mutual interaction of flow of aircraft elements, arising from the fact that they are produced by load-bearing elements of flow disruption, also deformed by non-load bearing elements (e.g., fuselages). They mutually distort generators of force, thus affecting back the flow fields, generated by them. In aircraft a crucial role is played by an interference between the main rotor (propeller) and a wing. A consequence of this phenomenon is usually a deterioration of aircraft flight parameters. In work [39], this phenomenon was defined as a phenomenon of a mutual interaction of various parts of the aircraft, as a result of which the aerodynamic force influencing the whole aircraft is not equal to a sum of aerodynamic forces affecting its isolated components, such as the wing, fuselage, stabilizer and so on. It is very important to properly shape the geometry of an aircraft. Special attention needs to be drawn to the place of joining the components (e.g., wings with the fuselage). A proper connection of the components allows for reducing interference drag force. In special cases, the aerodynamic drag of an entire aircraft can be smaller than the sum of aerodynamic drag of its isolated parts.

Research into rotors operating in autorotation is conducted worldwide. However, there is an insufficient number of studies on the interference of additional propulsion units upon the operation of the main rotor [40]. In the available literature one may find a range of works in the field of experimental and numerical aerodynamics, although they are mostly targeted at the gyroplane without a rotor. It should be noted that disregarding the impact of the main rotor and propellers originates from the fact that an examination of the rotor, extended in such a way, is extremely complex. The methodology of this type of research has been described in [41,42]. In work [4], the authors present experimental studies of the impact of the angle of horizontal stabilizers upon aerodynamic characteristics of a gyroplane. The object of research was a model of a gyroplane fuselage with H–tail stabilizer. The object allowed changing the stabilizer angle. However, the impact of the main rotor and driving propellers was disregarded. The fuselage with stabilizers were analyzed for the angle of incidence of the horizontal stabilizer within the range between −10° and +10° and for angles of attack in the range of −16° and 18°. For each analyzed configuration and an uninterrupted flow rate equal to 30 m/s, an aerodynamic load was designated. The obtained results show that a change in angle of incidence may cause both a quantitative and qualitative change in aerodynamic characteristics, which means that this parameter affects not only the expected performance of a gyroplane but also its longitudinal stability. The gyroplane of an incorrectly matched angle of incidence may be unstable, and thus dangerous to use. Aerodynamic calculation of impact of the main rotor on the fuselage and the tail of a light gyroplane were considered in work [43]. The CFD (Computational Fluid Dynamics) analysis of the complete gyroplane was made. Computation was performed for the model of gyroplane which was equipped with the two sub-models of the main rotor and the engine-powered propeller. Both of them were modelled as the actuator discs. The main goal of this research was to find the magnitude of the main rotor impact on the fuselage and the tail. To measure this effect, the main stability derivative changes of gyroplane body were investigated.

## 2. Research Object

**Figure 1:**
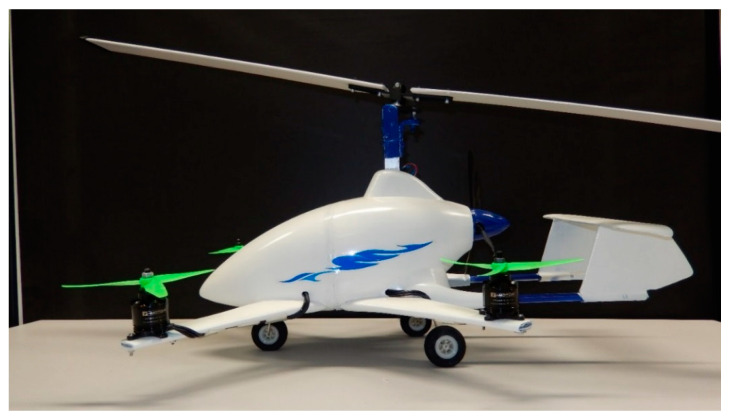
View of a complete aircraft with additional propulsion units—side view.

**Figure 2:**
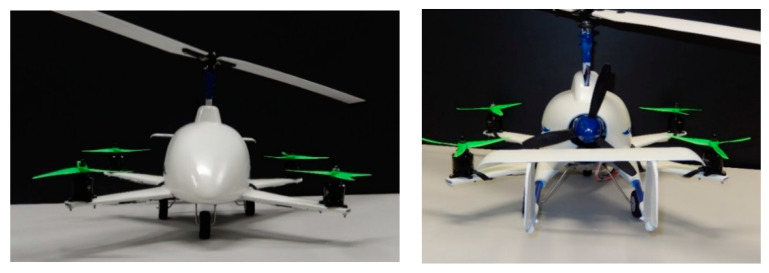
View of a complete aircraft with additional propulsion units—front view (on the **left**) and a rear view (on the **right**).

The authors’ own concept of the aircraft combines the advantages of a gyroplane (as a light and simple design, cheaper to operate) with a multirotor set, allowing a shorter take-off or a vertical take-off and landing, thus ensuring a stabilization of the flight at low speeds and an increase in its safety. A combination of these two aircraft will result in an innovative means of individual air transport, see an exemplary visualization in Figure 1 and Figure 2. The essence of a hybrid aircraft shown below (as an example of its manufacture) is the fact that it is composed of additional drive units generating thrust, mounted onto the fuselage. A progressive movement is possible by starting up a propulsion unit of a horizontal vector of the thrust force (pusher). There is also a version with an adjustable vector of thrust of two rear propellers but with no pusher. For the purpose of this work, it was decided to use the first configuration, forming a research platform to develop the issue of aerodynamic interference. An important feature of the device is the fact that it allows for reducing the take-off and landing of an aircraft. The device makes it possible to take off vertically or at any angle to the surface, which proves impossible in the case of other gyroplanes. The advantage is also an accurate and reduced landing, including a vertical one. The take-off and landing of the discussed aircraft are safe mainly due to additional units generating thrust, ensuring stability in all flight phases.

The test facility was performed in 3D printing technology. This type of technology is increasingly used in research and development in the airline industry, energy industry and many others [4,44,45,46]. The design process of the aircraft model started by printing a digital model that had been prepared beforehand, using powder-based 3D printing technology. The printouts were divided into three stages. In the first stage, the stabilizer was printed, in the second part—a fuselage, and finally the rear part of the fuselage. The preparation for printing itself consists in importing a file with a digital model and selecting an appropriate location of the printed part in the printer chamber. The device used for printing is ZPrinter 450 manufactured by 3D Systems.

## 3. Research Methodology

In the conducted aerodynamic aircraft investigations, several techniques have been used with regard to the measurement, computing flight properties and phenomena occurring during an airflow. The most recent research trend is a synergy of experimental tests in wind tunnels and numerical calculations. This research was carried out in the Institute of Aviation in Warsaw in a continuous-flow low speed wind tunnel with an open measuring space of 1.5 m in diameter and 2.0 m in length. The maximum speed of the air in the tunnel reached 40 m/s, whereas the minimum one was equal to 10 m/s. The turbulence intensity was limited to 0.5%. The stand is an element of the measurement and control tunnel, described in more detail in [47]. The force testing and visualization by means of image anemometry allowed for developing aerodynamic characteristics and defining a velocity field speed for the considered design configurations. A visualization of the flow field will be the subject of a separate article. Based on the conducted investigations, the authors made an analysis of findings in terms of a mutual impact of particular components of the research object and an area of impact of active elements, present in a common flow.

The experimental tests of the model were carried out on the FFA I-646-2 six-component internal balance (manufactured by the Institute of Aviation in Warsaw, Poland). The force balance was mounted on the bottom arms so the rear of the model was not modified. Before a series of experimental tests of the research model were conducted in the wind tunnel, the force balance was calibrated. The calibration performed to check the force balance showed the correct accuracy of its indications. Table 1 shows the statistics of force balance measurement error.

**Figure 3:**
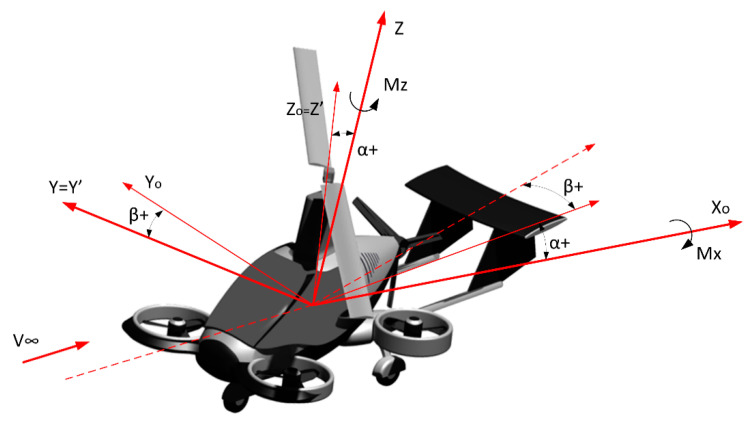
The adopted reference system associated with the flow.

Before the tests, the authors specified angles between airspeed and axes in the vertical plane and the horizontal plane. They also eliminated the impact of this factor on the measurements of aerodynamic characteristics. The reference system associated with the flow adopted in the research (in which forces and aerodynamic moments were measured), as well as the reference system associated with the model and the angles of rotation between these systems, are shown in Figure 3. Regardless of the angle of attack and the sideslip angle, the center of the reference system was located close to the axis of the wind tunnel.

For the calculations of aerodynamic coefficients, the following geometric data of the model were assumed:
● S = 0.9498 m^2^–  rotor disc area,● R = 0.55 m–  reference length to determine the coefficients of the moment of aerodynamic forces.

The center of the coordinate system was located on the plane of symmetry of the model (in the force balance axis). The distance between the center of the coordinate system and the center of force balance equaled 0.146 m.

## 4. Research Results

**Figure 4:**
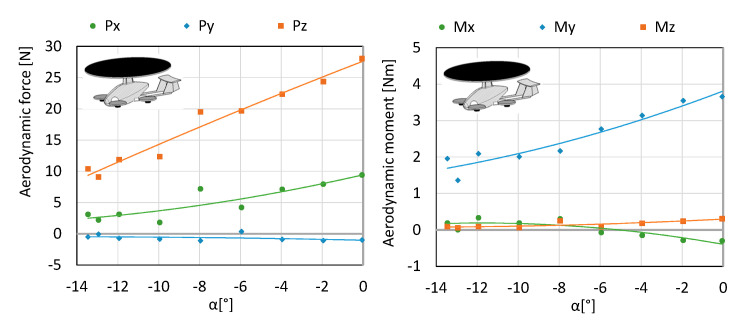
Characteristics of forces and moment of aerodynamic forces for configuration K1.

**Figure 5:**
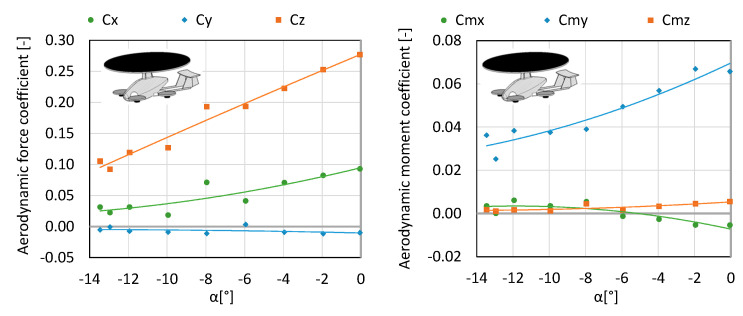
Characteristics of coefficients of forces and aerodynamic moment for configuration K1.

**Figure 6:**
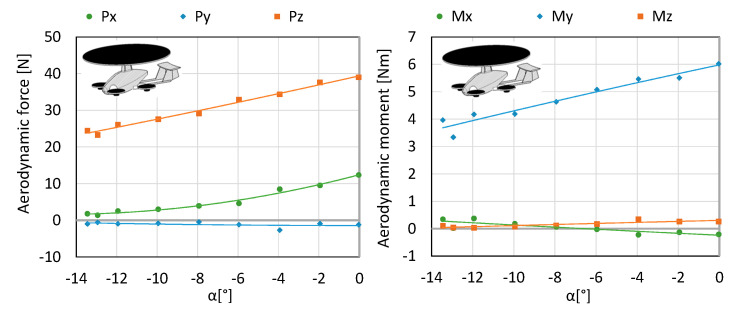
The characteristics of forces and moment for aerodynamic forces for configuration K2.

**Figure 7:**
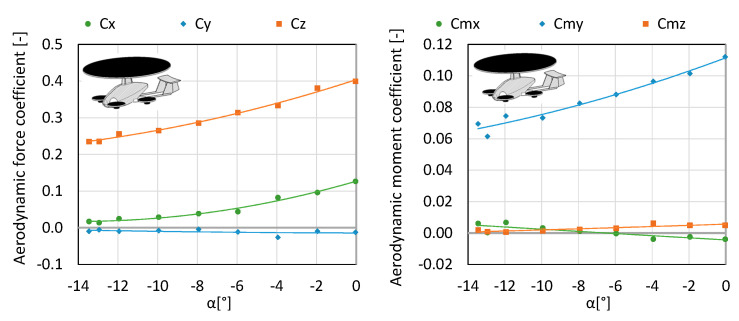
The characteristics of coefficients of forces and aerodynamic moment for configuration K2.

**Figure 8:**
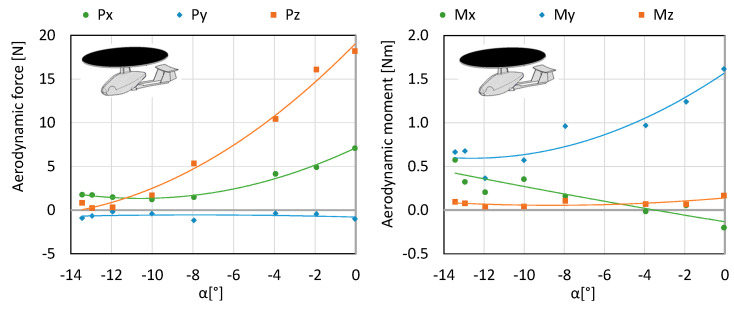
Characteristics of forces and moment of aerodynamic forces for configuration K3.

**Figure 9:**
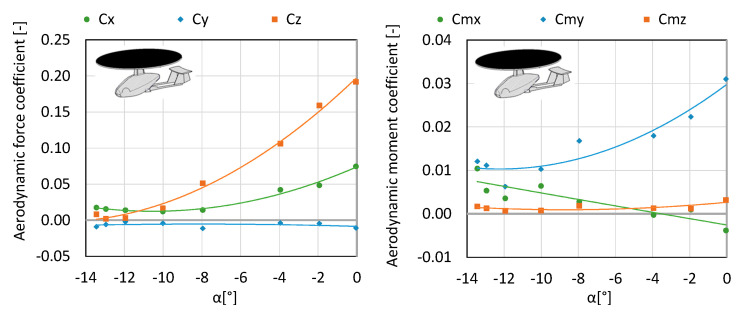
Characteristics of coefficients of forces and moment of aerodynamic forces for configuration K3.

For the needs of the analysis, three configurations were taken into account, marked accordingly as K1, K2 and K3. The drag force *Px*, lateral force *Py*, lift force *Pz*, roll moment *Mx*, pitch moment *My*, yaw moment *Mz* were analyzed. The results are shown in Figure 4, Figure 5, Figure 6, Figure 7, Figure 8 and Figure 9. Owing to the conducted measurement investigations, it was possible to obtain the maximum lift force of the rotor, permitted by the manufacturer, at a mounting angle equal to 13.4°. Therefore, in further measurements the rotor was mounted onto the fuselage at an angle of 13.5°. This means that when the fuselage is set at an angle of attack equal to 0°, the rotor operates at a maximum angle, i.e., 13.5°. It was assumed in the study that if the aircraft from the hover moves on to level flight (based on additional propellers generating the lift force), the nose of the fuselage goes down, i.e., it moves to the negative values of the angles (e.g., multicopter). For this reason, the tests were conducted for negative angles of attack. In the future, there are plans to develop a new configuration by eliminating the pusher propeller. The presented research object is an extended version. Configuration K1 consists of a fuselage, stabilizer, main rotor, arms, electric motors and propellers 10” in diameter. The configuration was tested with an air blast equal to 12.5 m/s in order to determine the aerodynamic forces influencing the aircraft and the examination of the impact of the angle of attack on the generated forces. The engines were started up at 50% of the available power range, whereas the main rotor itself operated in autorotation since flows were generated in the tunnel. The testing was conducted for a range of angles of attack from 0° to −13.5°. Angles of attack were calculated based on the position of the fuselage with respect to the undisturbed velocity vector in the symmetry plane of the test object. In the considered case, the rotor is attached in such a way that when the fuselage is set at an angle of attack 0°, the rotor is rigidly set at an angle of 13.5°. Therefore, it is clear that when the fuselage moves into negative angles, the angle of setting the main rotor is on the decrease. Within the considered angles of attack, the authors obtained thrust force ranging from 9 N to 28 N (see Figure 4 and Figure 5).

Configuration K2 consists of a fuselage, stabilizer, main rotor, arms, electric motors and propellers, 10” in diameter. The configuration was tested at an air flow equal to 12.5 m/s in order determine the aerodynamic forces acting on an aircraft and the examination of the impact of the angle of attack on the generated forces. The engines were started up at 100% of the available power range, while the main rotor operated in autorotation (since flow was generated in the tunnel). The testing was conducted for a range of angles of attack from 0 to −13.5°. Angles of attack were calculated based on the position of the fuselage with respect to the undisturbed velocity vector in the symmetry plane of the test object. Within the considered angles of attack, the lift force of 23.26–39 N (Figure 6 and Figure 7) was obtained.

Configuration K3 merely consists of the fuselage, stabilizer and main rotor. It was examined at an air flow equal to 12.5 m/s in order to determine the aerodynamic forces affecting the aircraft as well as investigating the impact of the angle of attack on the generated forces. The main rotor operated in autorotation, since it was possible to generate flow in the tunnel. The testing was conducted for a range of angles of attack from 0 to −13.5°. The angles of attack were calculated as in the previous configurations. Within the considered angles of attack, the authors obtained the lift force of 23.26–39 N (Figure 8 and Figure 9).

## 5. Analysis of Results

**Figure 10:**
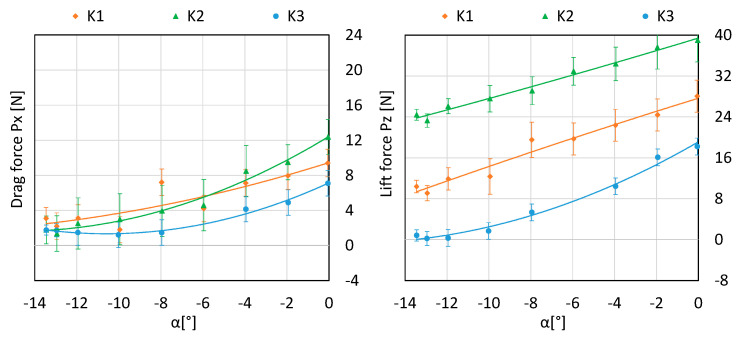
A juxtaposition of the impact of the angle of attack on the value of the drag force and lift for the three configurations under scrutiny.

Figure 10 shows a summary of the effect of the angle of attack on the value of the drag force and lift force for considered configurations.

The research that had already been carried out in the wind tunnel, aimed at measuring the forces and aerodynamic moments affecting the developed multirotor aircraft, underwent a statistical analysis. The proof of the importance of the impact of additional propellers on the operation of the main autorotation rotor and on the aerodynamic characteristics is by refuting a hypothesis of equality of mean values derived from two configurations of the research object (13):

(13) $$H_{0}=\mu_{1}-\mu_{1}=0$$

An analysis of measurement data included the calculation of average values of the considered measurement points and standard deviations (14) and (15):

(14) $$\bar{X}=\frac{1}{n}\overset{N}{\underset{i=1}{\sum }}X_{i}$$

(15) $$\sigma_{x}=\sqrt{\frac{\sum_{i=1}^{N}{\left[x\left(i\right)-\bar{X}\right]}^{2}}{n-1}}$$

In order to establish a proof, the Student’s t-test was applied. The subject of the verification was hypothesis *H*_0_ of mean values equality, derived from two series of measurements. For this reason, the value t1,x was computed, in accordance with the formula:

(16) $$t_{1,x}=\frac{\bar{X_{1}}-\bar{X_{2}}}{\sqrt{\frac{\sigma_{x1}^{2}}{n_{1}}+\frac{\sigma_{x2}^{2}}{n_{2}}}}$$

where: *x*—further configurations of an aircraft Kw.

Next, the level of confidence was calculated α for subsequent examined cases. The statistical significance of the obtained findings was based on the value of a significance level α. As a boundary value of the acceptable level of error, the level of confidence was accepted as *α* = 0.05 if:

- α<0.01 the difference is highly significant,
- 0.01 ≤ α<0.05 the difference is significant,
- 0.05 ≤ α<0.10 the difference is negligible,
- 0.10 ≤ α the difference is statistically insignificant.

Table 2 contains the findings of the significance of changes in the pitching moment *My* between configuration K3 and subsequent examined configurations.

Due to the testing for the direct flow of air in the tunnel and the placement of the research object at the angle of bank β = 0°, the greatest changes for the considered configurations are reported for the drag force *Px*, lift force *Pz* and the pitching moment My. Due to the symmetry of the object against the xz plane, the resultant lateral force *Py* is reduced to zero. Therefore, it was not analysed. It is also the case for the yawing moment *Mz*, which should also equal zero due to the fact that the main rotor has an additional degree of freedom. Because of the uneven loading of the main rotor operating in autorotation, changes occur in the rolling moment *Mx*. However, the largest absolute values of the forces act longwise on the x and z axis and the largest absolute values of the moment act with respect to the *y* axis.

## 6. Discussion and Conclusions

In the research, the authors formulated a thesis that assumes a significant impact of an additional multirotor propulsion unit which acts in the horizontal plane and generates lift by the main gyroplane rotor. In order to confirm this thesis, the values of lift, generated in three configurations, have been presented. The configuration K1 concerns the performance of the main rotor (uninterrupted by other active elements), the second only refers to the operation of additional propellers, while the third one involves both of them, involved in a mutual flow. The values of the thrust force generated by an additional multirotor propulsion unit which operates in the horizontal plane for 50% and 100% of the available power are equal to 12.63 N and 28.73 N, respectively. Besides, the main rotor operating in the tunnel (in autorotation) of the same flow rate generates the lift force equal to 18.2 N. Theoretically, without any interruptions or a mutual impact, a combination of these two systems should ensure a summative thrust force, in the first case, equal to 30.83 N (for power equal to 50%), and in the second one of 46.93 N (for power equal to 100%). Due to the aerodynamic interference, these values are equal to 28.02 N and 38.98 N, respectively, which is a reduction in the value of the lift force with reference to the theoretical value (resulting from a sum of forces generated by the system, with each force considered separately) by 9.1% and 16.9%, respectively. Being extreme values, however, in conditions of a proper operation of an aircraft, they become reduced because of a smaller demand for lift force. Assuming that the gravity force of an unmanned aerial vehicle is equal to the force generated by the main rotor carrier, it is definitely possible to reduce the need for power of a multirotor propulsion unit operating in a horizontal plane. Thus, a reduction of a negative impact of aerodynamic interference is anticipated.

## Figures and Tables

**Table 1:** Measurement error statistics.

	Px	Py	Pz	Mx	My	Mz
Average relative error (% of range)	−0.007	−0.001	−0.001	0.001	0.000	−0.001
Average relative error (% of current force)	0.131	0.467	0.114	0.072	0.122	0.422

**Table 2:** Test results of significance of changes in the drag force, lift force and pitching moment *My* among the considered configurations.

Angle of Attack α [°]	Drag Force Px		Lift Force Pz		Pitching Moment My	
	K1/K3	K2/K3	K1/K3	K2/K3	K1/K3	K2/K3
0	0.153	0.019	0.043	0.057	0.037	0.058
−2	0.060	0.001	0.029	0.058	0.045	0.058
−4	0.061	0.081	0.051	0.060	0.042	0.055
−6	-	-	-	-	-	-
−8	0.041	0.307	0.054	0.060	0.002	0.054
−10	0.693	0.453	0.042	0.060	0.025	0.053
−12	0.286	0.658	0.056	0.060	0.041	0.059
−13	0.695	0.811	0.057	0.060	0.153	0.057
−13.5	0.192	0.991	0.059	0.060	0.031	0.059
